# Uncovering a Role for Electronic Personal Health Records in Reducing Disparities in Sexually Transmitted Infection Rates Among Students at a Predominantly African American University: Mixed-Methods Study

**DOI:** 10.2196/medinform.9174

**Published:** 2018-07-12

**Authors:** Kevon-Mark Jackman, Stefan David Baral, Lisa Hightow-Weidman, Tonia Poteat

**Affiliations:** ^1^ Center for Public Health and Human Rights Department of Epidemiology Johns Hopkins Bloomberg School of Public Health Baltimore, MD United States; ^2^ Institute for Global Health and Infectious Diseases University of North Carolina at Chapel Hill Chapel Hill, NC United States

**Keywords:** PHR, STI, HIV, mHealth, intervention, prevention, young people, black, health disparities

## Abstract

**Background:**

Black youth continue to bear an overwhelming proportion of the United States sexually transmitted infection (STI) burden, including HIV. Several studies on web-based and mobile health (mHealth) STI interventions have focused on characterizing strategies to improve HIV-related prevention and treatment interventions, risk communication, and stigma among men who have sex with men (MSM), people who use substances, and adolescent populations. The Electronic Sexual Health Information Notification and Education (eSHINE) Study was an exploratory mixed-methods study among students at a historically black university exploring perceptions on facilitating STI testing conversations with partners using electronic personal health records (PHRs).

**Objective:**

The purpose of this paper is to use eSHINE Study results to describe perceived impacts of electronic PHRs on facilitating STI testing discussions between sexual partners.

**Methods:**

Semistructured focus groups and individual in-depth interviews were conducted on a heterogeneous sample of students (n=35) between May and July 2014. Qualitative phase findings guided development of an online survey instrument for quantitative phase data collection. Online surveys were conducted using a convenience sample of students (n=354) between January and May 2015. Online survey items collected demographic information, sexual behaviors, beliefs and practices surrounding STI testing communication between partners, and beliefs about the impact of electronic PHR access on facilitating these discussions with partners. Chi-square analysis was performed to assess gender differences across quantitative measures. A Wilcoxon signed rank sum test was used to test the null hypothesis that electronic PHRs are believed to have no effect on the timing of dyadic STI health communication.

**Results:**

Participants described multiple individual and dyadic-level factors that inhibit initiating discussions about STI testing and test results with partners. Electronic PHRs were believed to improve ability to initiate conversations and confidence in STI screening information shared by partners. Among online survey participants, men were more likely to believe electronic PHRs make it easier to facilitate STI talks with potential partners (59.9% vs 51.9%; χ^2^=3.93, *P*=.05). The Wilcoxon signed-rank test results indicate significant increases in perceived discussion timing before sex with electronic PHR access (61.0% vs 40.4%; *P*<.001).

**Conclusions:**

Findings suggest that electronic PHR access in STI screening settings among similar populations of Black youth may improve both motivation and personal agency for initiating dyadic STI health communication. Results from this study will likely inform novel interventions that use access to electronic PHRs to stimulate important health-related discussions between sexual partners. Moving forward requires studying strategies for implementing interventions that leverage electronic PHRs to create new sexual health communication channels with providers, peers, and family among black youth.

## Introduction

### The High Burden of Sexually Transmitted Infections Among Black Youth and Dyadic Sexually Transmitted Infection Testing Talks

Young black people in the United States (US) are largely overrepresented in cases of sexually transmitted infections (STIs), including HIV. While black people constitute approximately 15.4% of US youth ages 15 to 24 years, they accounted for 29.9% of chlamydia cases, 47.7% of gonorrhea cases, 43.3% of early syphilis, and 54.7% of HIV cases diagnosed in this age group in 2016 [1-3]. Getting young people to talk about STI testing, including HIV, with partners prior to sex is a critical challenge to disease prevention and control [4]. These preventative conversations support testing, disease status disclosure, condom use, and the use of medicines to prevent and treat STIs [5]. The “Get Yourself Tested” campaign launched nationally in 2009 in effort to reduce STIs in young people by targeting four key behaviors: STI testing, HIV testing, talking to partners about testing, and talking to providers about testing. Formative research used to inform the campaign design found that STIs remain highly stigmatized and as a result, few young people discuss testing with partners prior to sex [4,6].

In young people, conversations on STI testing are more likely to be facilitated by those that routinely screen for STIs [7]. In studies of students at historically black colleges and universities, women reported higher screening rates and asked partners about HIV status more than their male counterparts [8,9]. Additionally, individuals are more likely to discuss STI status and risk behaviors with a main partner compared to a casual partner [10]. Expected partner reactions of suspicion, accusation, or insult (from inadvertently implying partner distrust) are sometimes barriers to discussing STI testing [4]. Barriers that inhibit partner communication regarding STI risk restricts the ability to make well-informed decisions about the level of risk assumed and employable methods to prevent disease transmission [11,12].

### Digital Prevention Tools

The US National HIV Strategy calls for federal agencies to encourage the development and implementation of highly accessible digital tools to educate and inform the American people with scientifically accurate information on disease risk, prevention, transmission, and treatment [13]. Several studies on Web-based and mobile health (mHealth) HIV/STI interventions have been conducted and are currently ongoing that focus on improving outcomes related to testing, risk communication, reducing stigma, increasing condom use, and improving adherence to biomedical prevention and treatment among men who have sex with men (MSM), substance using, and adolescent populations [14-19]. Although mHealth intervention studies focused on improving partner communication on STI testing are promising, most studies do not account for the role of remote access to STI screening records in dyadic STI health communication. Electronic personal health records (PHRs) or patient portal services (eg, Epic MyChart, Cerner Patient Portal, Kaiser Permanente My Health Manager, Quest Diagnostics Care360) provide patients with remote access to their laboratory test results, including STI results. Electronic PHRs differ from most mHealth platforms in STI intervention studies since electronic PHRs contain protected health information managed by covered entities, therefore, federal regulations require electronic PHR vendors to adhere to data security and integrity measures outlined by the Secretary of the Department of Health and Human Services [20]. Nevertheless, electronic PHRs can provide several services in addition to medical record access, like educational resources and tools for communicating with healthcare providers.

### Study Purpose

The Electronic Sexual Health Information Notification and Education (eSHINE) Study was a mixed-methods study among students 18 to 25 years old attending a historically black university. The project was completed using a dissertation research funding grant from the Agency for Healthcare Research and Quality, and explores perceptions of using electronic PHRs to share electronic STI screening information between sexual partners. The current study is an analysis of eSHINE Study qualitative and quantitative data to explore attitudes and practices surrounding STI testing talks between partners, barriers to talking about STI testing with partners, and perceived impacts of electronic PHRs on risk discussion facilitation. Thus, the purpose of our study is to provide a rich contextual understanding of how diffusing electronic PHR access in our study population may impact health-related communication between sexual partners.

## Methods

### Study Design

Exploratory mixed methods are a two-phase sequential study design that is particularly useful for exploring new research questions [21]. It calls for an initial qualitative exploration phase (ie, focus-groups and individual interviews), an intermediate survey development phase, and a second quantitative research phase (ie, an online survey) [22]. A constructivist approach of incorporating multiple theoretical frameworks structured our study protocol. The Integrative Model of Behavioral Prediction (IMBP), often used for designing health messages, posits that behavioral intentions are a function of attitude, normative, and self-efficacy beliefs [23]*.* IMBP also considers the impact of demographic variables, culture, and environmental constraints on behavior. If electronic PHR–facilitated STI talks is a novel concept to the study population, constructs of the Diffusion of Innovation Theory (DOI), such as innovation attributes and communication channels, were incorporated into our exploration [24]. Finally, the Disclosure Processes Model provide considerations for individuals with a history of STI diagnosis or exposure; here, disclosing risk is a function of antecedent goals (ie, approach goals vs avoidance goals) [25].

### Recruitment

Eligible study participants were students, ages 18 to 25 years, enrolled at a southern historically black college and university at the time of study. Qualitative phase participants were recruited between May and July 2014 and quantitative phase participants between January and May 2015. Recruitment flyers were posted on campus along with multiple announcements sent through the university’s student, faculty, and staff email list. Targeted recruitment efforts were conducted in collaboration with University Health Services, the Student Counseling Center, the Office for Residence Life to post study materials and conduct tabling events. Study flyers were posted to a university affiliated Facebook page for lesbian, gay, bisexual, transgendered, and questioning students. Participants were also recruited at student organizational meetings, including theatre, peer educator, and football. Study enrollment included providing eligible students with a detailed description of the project, participation requirements, and terms for receiving incentives. To complete enrollment, prospective participants signed an informed consent form in person or online using Adobe Echosign. Qualitative and quantitative phase research protocols, including focus-group, interview guides and online survey instruments were separately reviewed and approved by the university’s Institutional Review Board.

Qualitative phase participants received US $25 cash for each session (limited to one focus group or one interview per participant). Quantitative phase participants received US $20 cash for completing the online survey; and if eligible, qualitative phase participants could participate in the quantitative phase. Online survey participants were not asked to provide their name or email address on the survey. To receive incentives, participants provided a unique code generated at survey completion. Participants were additionally entered to win prizes in one of three raffle drawings (eg, textbook vouchers, US $25 up to US $100 gift cards, Samsung Galaxy tablets). eSHINE Study data collection and analyses was conducted by KJ as part of his dissertation research, with guidance from project mentors and dissertation committee members. Prior to the study, KJ completed training in qualitative and quantitative research methods, including academic coursework and work as an STD prevention and control program disease intervention specialist.

### Qualitative Methods

A total of 35 students participated in the qualitative research phase (19 male; 16 female). Audio-recorded focus group and individual interview sessions were conducted by KJ inside private conference rooms located on the university’s campus. In May 2014, 33 students participated in one of three separate focus group sessions (n=6; n=10; n=17). Semistructured sessions averaging 70 minutes in length were divided into three discussion sections: (1) electronic PHR perceptions, (2) experiences and perceptions related to dyadic STI health communication, and (3) perceptions related to using electronic PHRs in dyadic STI health communication. At the end of each focus group, participants were invited to schedule an in-depth individual interview session. Focus group and interview recordings were played back after sessions by KJ to construct field notes and inform any modifications to main questions in subsequent sessions.

 

Semistructured interviews were important to explore in depth the statements made by participants during focus groups in a setting isolated from peers. On average, interviews lasted 45 minutes. An oral questionnaire was administered during interviews to collect demographic information, orientation, and sexual risk behavior, such as number of recent sex partners, condom usage, and sex under the influence of alcohol or drugs practices. Considering the sensitivity of sexual topics and to support higher levels of comfort among participants, this information was not collected during focus groups. Sixteen of eighteen individual in-depth interviewees were recruited from a focus group session. Transcripts and field notes were uploaded to ATLAS.ti to facilitate the qualitative analysis [26]. The analysis included reading through transcripts and field notes while identifying useful quotes or sentences, creating memos, coding segments of information, assigning labels to codes, and the grouping of codes into broad themes [21]. 

### Survey Development

A Qualtrics online survey was developed during the intermediate survey development phase between July and December 2014 [27]. Qualitative codes were operationalized into IMBP behavioral construct variables, such as attitudinal, self-efficacy, and intentional beliefs within broad emergent themes. The operationalization process included using individual codes within themes as variables as well as using specific quotes from participants within items on the survey [21]. Behavioral construct variables, for example, attitudes on the importance of discussing STI testing with partners, were measured using 7-point Likert scale items, scored ­–­3 to 3 [28]. Data was also collected on several demographic and sexual behavior variables, such as gender, screening practices, and risky sex behaviors. The survey was piloted with eight students and revisions made based upon participant feedback and researcher observations. The final survey had 116 items and a completion time of approximately 30-45 minutes.

### Quantitative Methods

To access the online survey, a secured and unique Web-link was sent to the enrolled participant’s student email account using the university’s email database. Between January and May 2015, 1093 participants registered for the online survey and were emailed secured survey links; 501 surveys were started, 380 completed, and 354 completed without missing data.

Survey data were uploaded into STATA 14 for statistical analyses [29]. Chi-squared analyses were conducted by gender to describe distributions of demographic information, risk behaviors, STI testing talk attitudes and practices, and perceptions of electronic PHR–facilitated discussions by gender. Effect size (Cohen *d*) was calculated for variables demonstrating significant differences by gender related to electronic PHR impacts on discussions. A Wilcoxon signed rank sum test was performed to determine differences in perceived discussion timing with electronic PHR access compared to without electronic PHR access. To test the null hypothesis that electronic PHRs are believed to have no effect on the timing of STI conversations, we compared responses on two survey items: (1) “When do discussions about STD testing typically occur between you and your partner(s)?” and (2) “When would discussions about STD testing likely occur if you and your partner(s) had electronic PHRs?” Reponses were ranked from 1 to 4; (1) never (2) after sex (3) inconsistently before or after sex, and (4) before sex.

## Results

### Qualitative Phase Results

Qualitative phase participants were heterogeneous in academic classification, degree major, sexual behaviors, sexual orientation, including for example, student athletes, peer educators, and members of Greek lettered social organizations. Table 1 presents interview and focus group quotations from our qualitative phase study. For this study, quotations were categorized under two major themes: attitudes and practices surrounding dyadic STI health communication and expected impacts of electronic PHRs on dyadic STI health communication. Table 1 also indicates online survey measures derived from qualitative codes.

### Sexually Transmitted Infection Testing Talks: Qualitative Attitudes and Practices

STI testing talks or risk discussion events were described as verbal exchanges between sexual partners regarding STI testing or status. Soliciting information related to STI risk from partners was described as demonstrating personal responsibility and an unalienable right to self-preservation:

You have the right to know.

I feel like if you’re having sex with me you have the right to know my STD history. If I’m having sex with you, I need to know everything too. I owe that to you, you owe that to me.

Conversations vary in both timing and depth. Several multi-level factors, such as self-efficacy, partner-type, and intoxication, can impact when and how discussions occur (Table 1). When talks occur, they are either distal, proximal, or after the sexual encounter. Distal discussions happen in a period where individuals are “getting to know each other.” While proximal discussions occur when participants are “in the moment.” Discussions can range in depth from simply asking, “are you good down there?” to requiring current screening information and information regarding condom use practices with previous partners.

Valuation for risk discussions varied between participants. For most, STI talks are very important to always occur, especially interviewees disclosing a history of STI infection.

It’s very important to have that conversation.

Some participants saw no purpose in talking about STI testing when using condoms. Valuation also varied based on dyadic characteristics such as, partner type.

If I know that I am going to be in something committed then I want to know your history. But if you are just a casual partner then I don’t really care, because I am going to protect myself. I wouldn’t have the conversation with someone I’m just casually having sex with.

Participants described sexual partner dynamics ranging from solely pleasure-seeking sex and noncommittal partnerships to socio or emotional interdependent and committed relationships. To simplify and operationalize, partner types were classified as: (1) main partners, defined as partnerships intended to be exclusive relationships; (2) casual partners, defined as recurring partnerships not intended to be exclusive relationships; and (3) hook-up partners, defined as one-time partnerships. Keeping in mind that dyad characteristics vary within partner-type classifications (for example, casual partners may be long-time friends or recent acquaintances, and others).

Finally, valuation appears to also be determined by the extent to which individuals are aware of the importance of discussing STI testing with partners.

I have never discussed STDs with any of my sexual partners. I’m young, so it never really came up. I usually just say, what’s the number of people you have had sex with and if I feel comfortable with the number then, okay.

### Barriers to Talking About Sexually Transmitted Infection Testing With Partners: Attitudes and Personal Agency

Self-efficacy contributes to whether STI testing talks are initiated. While some participants described being very comfortable with initiating conversations, many generally describe it as an “[i]t’s an awkward conversation.” The impact of these beliefs as an inhibitor is based on the level of disruption an individual believes the conversation will cause to a potential sexual encounter or relationship. Low self-efficacy to facilitate risk discussions was mostly described in the context of proximal discussions, where consequences of these events may “ruin the mood.” Lack of self-efficacy to initiate STI testing talks without ruining the mood presents as a primary barrier to some.

**Table 1:** Focus-group and individual in-depth interview quotations and online survey measures derived from qualitative codes. PHR: personal health record; STI: sexually transmitted infection.

Themes and online survey measure		Quotations	
**Attitudes and practices surrounding dyadic STI health communication**			
	Timing of dyadic STI health communication	*It usually occurs before anything else. After I get to know the person, it comes up in the conversation because this person is a potential partner. I always ask to be on the safe side.* *Before I have sex, we don’t have the conversation. It slips my mind until after we have sex.*	
	Valuation for dyadic STI health communication	*It is very important to have that conversation, especially if you are going to be dealing with that person sexually, “Have you been tested?” “Are you going to get tested?” “When was the last time you were tested?*	
	Valuation for dyadic STI communication when using condoms; communications barrier: condom use	*If I know that I am going to be in something committed, then I want to know your history. But if you are just a casual partner then I don’t really care, because I am going to protect myself.*	
	Self-efficacy to initiate dyadic STI health communication	*It doesn’t make me uncomfortable, I am straightforward. If I feel that we are about to get serious or have any sexual encounters, I simply ask “when was the last time you got tested?” If it’s too long, I tell them where to go for testing.*	
	Communication barrier: precontemplation	*When I was fresh out of high school, out of my parent’s house, I was [sleeping with] 6 or 7 guys at the same time. I was young, so I did not think to ask, have you gotten tested, how many people have you been with?* *I have never discussed STDs with any of my sexual partners.*	
	Communication barrier: awkward	*It kills the mood.* *(It can be awkward) when you have known the person.*	
	Communication barrier: people lie	*People lie. One of the big lies is “I’ve been tested” or “I don’t have anything.” Especially when you’re in the moment, it happens all the time.*	
**Expected impacts of electronic PHRs on Dyadic STI Health Communication**			
	More confidence in STI testing information shared by a partner	*It’s another way of verifying the truth and showing that they did get tested or if we need to get tested—we can go [to a test site] together.*	
	Easier for potential partners to talk about STI testing; easier check-in talks with partners on STI testing		*Ultimately the app would make it much easier to have these conversations with someone you are going to have sex with—whether it’s casual or long term.* *I can just show my partners casually when I got tested and my results. It will ease the tension and make it more comfortable, especially if I am willing to share that information with you.*
	Impact on frequency of STI talks; earlier STI talks (proximity to potential encounter)	*If the norm was for people to have the app at hand, then more people would ask to see results. Now, it’s not that realistic, because people can easily say, I don’t have it with me, it’s on paper.* *If the app is popular, then I’m asking everybody.*	
	Intentional beliefs to only use electronic PHRs when distrusting of partners	*I wouldn’t have a need for it, but then again if I do want to, you shouldn’t be offended, because I’m just trying to be safe. So, if I do bring it up, don’t be upset because it’s good health.*	
	Soliciting a partner’s electronic record will be awkward	*If I tell you something and you don’t believe it, we shouldn’t be having sex in the first place. If I tell you something, that’s what you should believe. If I am lying, then strap up [use a condom].* *It’s tricky, it’s one thing to ask someone something, but then to tell them to verify it, it messes up the trust. Unless if they are very comfortable.*	
	Self-efficacy for sharing a positive STI electronic PHR; preferred method to share STI positive status	*People who are negative would gladly show their results. People who are positive, it would be harder for them.* *I don’t see anyone showing a partner the app unless if they are clean.*	
	Suspicious of partners unwilling to share electronic PHR	*Red flag.*	

Some participants described talking with partners about STI testing as an exercise in futility because of limitations in the ability to verify shared information.

People lie. One of the big lies is “I’ve been tested” or “I don’t have anything”. Especially when you’re in the moment, it happens all the time.

Allaying a partner’s STI transmission concerns can take precedence over communicating accurate information about STI screening and risk. Several approaches are employed to mitigate risk associated with receiving inaccurate information. For example, some participants reported choosing to avoid risk discussions all together and use condoms. Conversely, some participants said that they engaged in couples testing or require potential partners show verification of STI test results prior to sex.

Any boy I ever ask that question to, I make sure I see papers. Paper says clearly negative or positive.

Though some participants commented that it is unlikely for “papers” to be readily available to validate STI screening information.

Other barriers exist to starting dyadic talks on STI testing. Substance use prior to sex was considered to inhibit ability for facilitating risk discussions with partners.

If someone is drunk, then it’s not going to be discussed.

Self-efficacy to initiate risk discussions may also be diminished when dyads have a previously established intimate relationship or a friendship. Difficulty discussing STI testing “when you have known the person” was described by participants as implying distrust.

### Impact of Electronic Personal Health Records on Sexually Transmitted Infection Testing Talks: Beliefs and Expectations

Information verification was considered the foremost benefit of incorporating electronic PHRs into risk discussions between sexual partners.

It’s another way of verifying the truth and showing that they did get tested or if we need to get tested— we can go (to a test site) together.

Participants used terms such as, “truth-detector,” “proof,” and “confirmation,” in referring to electronic PHR use with partners. Participants also described limitations of electronic PHRs in determining a potential partner’s real-time infection status.

There is no way to say you are clean today, but you can say you were clean that day.

Together, electronic PHRs are believed to be a compatible innovation for adding assurance to STI talks.

I think this will be something good for the gay community. The gay community is big on electronic dating and meeting people online, Grindr and Jack'd and all that. I feel that it would be really good for that.

Gaining electronic PHR access was believed to ease the ability to facilitate conversations on STI testing.

I can just show my partners casually when I got tested and my results. It will ease the tension and make it more comfortable, especially if I am willing to share that information with you.

Another participant explained:

Ultimately the app would make it much easier to have these conversations with someone you are going to have sex with—whether it’s casual or long term.

Participants added that electronic PHRs could make it easier to have “check-in” conversations with partners who have prior established intimate relationships or friendships. Some participants maintained that practices will be determined by partner-type.

If I have a one-night stand, I will use protection. I would not want to ask that question.

However, for most, the idea of an easier STI talk eliminates partner-type related factors as a barrier.

If the app is popular then I’m asking everybody.

Improvements to personal agency was not anticipated across some constraining conditions, such as intoxication or being infected with an STI.

If you’re drunk, I don’t think people would use it, because you really wouldn’t be thinking about that, your mind is somewhere else.

Similarly, it was expressed that individuals with electronic records positive for STI infection may employ strategies to avoid talking with partners about STI screening records.

People who are negative would gladly show their results. People who are positive, it would be harder for them.

Nevertheless, some participants believed that incorporating educational resources within electronic PHR products might prove useful in explaining positive test results and prevention.

While electronic PHRs were compatible with most participants as a potential tool for facilitating STI talks, some participants suggest electronic PHR solicitation as intrusive or implying distrust.

It’s tricky, it’s one thing to ask someone something, but then to tell them to verify it, it messes up the trust. Unless if they are very comfortable.

Additionally, partners unwilling to share electronic screening records are anticipated to raise a “red flag” regarding future sexual decisions and relationship progression. Overall, electronic PHR access was anticipated to have the population-level impact of increasing discussions on STI testing.

If the norm was for people to have the app at hand, then more people would ask to see results. Now, it’s not that realistic, because people can easily say, I don’t have it with me; it’s on paper.

### Quantitative Phase Results

Table 2 shows demographic information and sexual risk behaviors among online survey participants (n=354). The sample consisted of 167 male and 187 female participants. Forty out of 354 participants (11.3%) reported no history of sexual intercourse and 44.3% reported 2-5 partners. Approximately 43.2% (153/354) reported STI testing seven months prior to the study; 80 out of 354 participants with sexual exposure reported no history of screening (22.1%). Almost half (172/354) of participants reported sex under the influence of alcohol or drugs and 134 out of 354 participants reported recent sex without discussing STI testing (Table 2). Sixty-four percent of participants (38/59) with a history of STI diagnosis reported chlamydia infection. Additionally, five participants reporting gonorrhea, two participants reporting HSV-2, and two participants reporting human papilloma virus also reported chlamydia infections, there was no overlap between any other STIs (not shown).

### Sexually Transmitted Infection Testing Talks: Quantitative Attitudes and Practices

Table 3 presents behavioral perceptions on risk discussion practices among online survey participants. Conversations consistently occur before sex for 143 out of 354 participants (40.4%) and inconsistently occur before or after a sexual encounter for 41.0% (145/354). Some participants (51/354) reported never discussing STI testing with partners. Most participants (312/354) value discussing STI testing with partners, however, valuation was slightly greater among women (χ^2^=3.79; *P*=.05). In fact, 71.7% (254/354) believed it is extremely important or very important irrespective of condom-use, however over two-fifths (158/354) reported recent sexual encounters where risk discussions were skipped due to condom-use. Furthermore, less than half (169/354) believe that it is easy to talk with partners about STI testing and 28.2% (100/354) reported skipping a recent discussion due to awkwardness. One-third of participants (118/354) reported recently skipping risk discussions due to the possibility of receiving inaccurate information. Many participants (112/354) also reported discussion omission because it never came to mind.

### Impact of Electronic Personal Health Records on Discussion Timing

**Figure 1:**
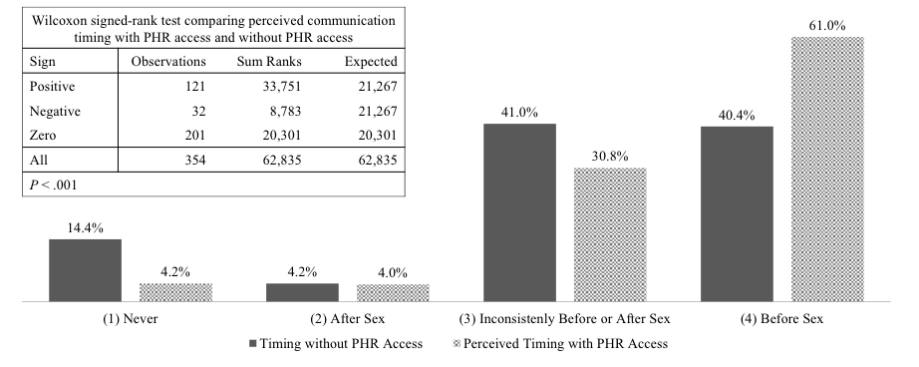
Perceived sexually transmitted infection communication timing with sexual partners with and without electronic personal health record (PHR) access among eSHINE Study online survey participants (n=354). The Wilcoxon signed-rank test indicates significant increases in perceived discussion timing before sex with electronic PHR access (*P*< .001).

Out of 354 online survey participants, 184 (60.0%) believed that electronic PHR access will lead sexual partners to start conversations on STI testing earlier in the relationship (Table 4). The Wilcoxon signed-rank test results shown in Figure 1 indicate significant differences between perceived discussion timing with and without electronic PHRs (*P*<.001). Discussions occurring before sex increased 20.6% with electronic PHR access; while omitting talks and inconsistent timing both decreased by 10% (Figure 1).

### Impact of Electronic Personal Health Records on Personal Agency, Information Assurance, and Assessing Partner Risk

Table 4 presents bivariate relationships between perceptions on incorporating electronic PHRs into risk discussion events and gender. Almost two-thirds (225/354) of participants felt that electronic PHRs would help to improve communication between partners about STI prevention. Similarly, most participants (235/354) believed electronic PHRs would increase their confidence in STI testing information shared by a partner. Electronic PHRs are believed to make it easier to facilitate risk discussions between new potential partners (197/354) and ongoing sexual partners (195/354). Nearly a quarter of participants (85/354) believed soliciting a partner’s electronic record would be awkward. Furthermore, two-fifths (154/354) believed it would be difficult to share a positive result with a partner. In fact, more participants (158/354) indicated a conversation without electronic PHRs as the preferred method for STI disclosure compared to using electronic PHRs (81/354). The most commonly held belief was that participants will be suspicious of potential partners unwilling to share electronic PHRs (268/354).

### Electronic Personal Health Record–Facilitated Sexually Transmitted Infection Talks: Beliefs by Gender

Male participants were more likely to believe that electronic PHR access would make it easier for sexual partners to discuss STI testing compared to female participants (59.9% vs. 51.9%; χ^2^=3.93; *P*=.05). However, the effect size by gender was small (*d*=0.27). Gender differences between beliefs that electronic PHR facilitated conservations would be awkward and self-efficacy beliefs for sharing electronic STI records with positive results had larger effect sizes. A smaller proportion of males (18.0% vs 29.4%) consider electronic PHR–facilitated discussions to be potentially awkward and were additionally more confident in their ability to share a STI-positive electronic result compared to female participants [(χ^2^=10.85; *P*=.001; *d*=1.04) and (χ^2^=6.48, *P*=.01; *d=* 0.80)].

**Table 2:** Demographic information and sexual risk behaviors among eSHINE Study online survey participants (n=354). IQR: interquartile range; STI: sexually transmitted infection.

Variables			Total, n (%)		Men, n (%)		Women, n (%)		Chi-square		*P* value
**Age**									—		—
	Median age (IQR)	20 (19-22)		20 (19-22)		20 (19-22)					
**Academic classification**									23.64		<.001
	Freshman	89 (25.1)		57 (34.1)		32 (17.1)					
	Sophomore	82 (23.1)		42 (25.1)		40 (21.4)					
	Junior	87 (25.6)		37 (22.2)		50 (26.7)					
	Senior	88 (24.9)		31 (18.6)		57 (30.5)					
	Graduate student	8 (2.3)		0 (0.0)		8 (4.3)					
**Sexual preference by gender**									267.15		<.001
	Men only	172 48.6)		10 (6.0)		162 (86.6)					
	Women only	156 (44.1)		149 (89.2)		7 (3.8)					
	Men and women	26 (7.3)		8 (4.8)		18 (9.6)					
**Reported sex partners (in 12 months prior)**									18.88		<.001
	No partners in 12 months prior to study or no history of sexual intercourse	56 (15.8)		34 (20.4)		22 (11.8)					
	1	116 (32.8)		45 (26.9)		71 (38.0)					
	2	79 (22.3)		27 (16.2)		52 (27.8)					
	3-5	78 (22.0)		47 (28.1)		31 (16.6)					
	6+	25 (7.1)		14 (8.4)		11 (5.9)					
**Reported partner-types^a^**									—		—
	Main partner(s)	213 (60.2)		84 (50.3)		129 (69.0)					
	Casual partner(s)	153 (43.2)		77 (46.1)		76 (40.6)					
	Hook-up partner(s)	72 (20.3)		47 (28.1)		25 (13.4)					
**STI screening history**									21.14		<.001
	< 7 months	153 (43.2)		53 (31.7)		100 (53.5)					
	≥ 7 months	81 (22.9)		39 (23.3)		42 (22.5)					
	Never tested	80 (22.6)		51 (30.5)		29 (15.5)					
	No history of sexual intercourse	40 (11.3)		24 (14.4)		16 (8.6)					
**STI diagnosis history and risky behaviors in 12 months prior to study**											
	History of STI diagnosis	59 (16.7)		14 (8.4)		45 (24.1)		15.62		<.001	
	Concurrent sexual partners	68 (19.2)		38 (22.8)		30 (16.0)		2.56		.11	
	Sex under the influence of drugs or alcohol	172 (48.6)		65 (38.9)		107 (57.2)		11.82		.001	
	Condom-less sex with a casual partner	106 (30.8)		44 (26.3)		65 (34.8)		2.92		.09	
	Condom-less sex with a hook-up/one-time partner	26 (7.3)		12 (7.2)		14 (7.5)		0.01		.91	
	Met sex partners using social websites or applications	56 (15.8)		39 (23.4)		17 (9.1)		13.47		<.001	
	Sex without discussing STI testing	134 (37.8)		62 (46.3)		72 (38.5)		0.07		.79	

^a^Partner type categories reported by participants are not mutually exclusive.

**Table 3:** Behavioral attitudes and practices related to dyadic conversations on sexually transmitted infection (STI) testing among eSHINE Study online survey participants, bivariate analyses by gender (n=354).

Variables		Total, n (%)	Men, n (%)	Women, n (%)	Chi-square	*P* value
**Valuation belief for talking about STIs with partners (n=315)**					3.79	.05
	Very important/important	312 (88.1)	137 (82.0)	175 (93.6)		
	Very unimportant/unimportant	3 (0.8)	3 (1.8)	0 (0.0)		
**Valuation belief for talking about STIs with partners when using condoms (n=263)**					2.29	.13
	Very important/important	254 (71.7)	105 (62.9)	149 (79.7)		
	Very unimportant/unimportant	9 (2.5)	6 (3.6)	3 (1.6)		
**Self-efficacy belief to initiate risk discussions (n=213)**					0.00	.95
	Very easy/easy	169 (47.7)	76 (45.5)	93 (49.7)		
	Very difficult/difficult	44 (12.4)	20 (12.0)	24 (12.8)		
**Intentional belief on likelihood to solicit STI screening from a partner (n=254)**					1.57	.21
	Very likely/likely	226 (63.8)	93 (55.7)	133 (71.1)		
	Very unlikely/unlikely	28 (7.9)	15 (9.0)	13 (6.9)		
**Timing of STI testing talks with partners discussion (n=354)**					2.77	.43
	Before sex	143 (40.4)	68 (40.7)	75 (40.1)		
	Sometimes before sex and sometimes after sex	145 (41.0)	64 (38.3)	81 (43.3)		
	After sex	15 (4.2)	6 (3.6)	9 (4.8)		
	Never	51 (14.4)	29 (17.4)	22 (11.8)		
**Reasons for omitting dyadic STI health communication for sexual encounters in 12 months prior to study (n=354)**						
	Condoms were being used	158 (44.6)	86 (51.5)	72 (38.5)	0.00	.97
	The topic would make things awkward	100 (28.2)	47 (28.1)	53 (28.3)	0.14	.71
	People can lie about it regardless	118 (33.3)	54 (32.3)	64 (34.2)	6.03	.01
	The topic never came to mind	112 (31.6)	58 (34.7)	54 (28.9)	1.40	.24

**Table 4:** Perceptions on incorporating personal health records (PHRs) into risk discussion events among eSHINE Study online survey participants (n=354). STI: sexually transmitted infection.

Variables		Total, n (%)	Men, n (%)	Women, n (%)	Chi-square	*P* value
**Semantic belief on the effect of electronic STI record access on communication on STIs between partners (n=227)^a^**					1.74	.19
	Very helpful/helpful	225 (63.6)	105 (62.9)	120 (64.2)		
	Very harmful/harmful	2 (0.6)	0 (0.0)	2 (1.2)		
**Semantic belief on the effect of electronic STI record access on confidence in STI testing information shared by a partner (n=238)^a^**					0.19	.66
	Very helpful/helpful	235 (66.4)	108 (64.7)	127 (67.9)		
	Very harmful/harmful	3 (0.9)	1 (0.6)	2 (1.1)		
**Belief that electronic PHRs make it easier for potential partners to talk about STI testing (n=213)^a^**					3.93	.05
	Strongly agree/agree	197 (55 .6)	100 (59.9)	97 (51.9)		
	Strongly disagree/disagree	16 (4.5)	4 (2.4)	12 (6.4)		
**Belief that electronic PHRs make it easier to check-in with partners on STI testing and prevention (n=206)^a^**					0.53	.46
	Strongly agree/agree	195 (55.1)	93 (55.7)	102 (54.5)		
	Strongly disagree/disagree	11 (3.1)	4 (2.4)	7 (3.7)		
**Belief that soliciting a partner’s electronic record will be awkward (n=171)^a^**					10.85	.001
	Strongly agree/agree	85 (24.0)	30 (18.0)	55 (29.4)		
	Strongly disagree/disagree	86 (24.3)	52 (31.1)	34 (18.2)		
**Self-efficacy belief for sharing a positive electronic STI record with a partner (n=213)^a^**					6.48	.01
	Very easy/easy	59 (16.7)	36 (21.6)	23 (12.3)		
	Very difficult/difficult	154 (43.5)	64 (38.3)	90 (48.1)		
**Belief that partners using electronic PHRs will start talking about STI prevention earlier in a relationship (n=200)^a^**					0.70	.40
	Strongly agree/agree	184 (52.0)	89 (53.3)	95 (50.8)		
	Strongly disagree/disagree	16 (4.5)	6 (3.6)	10 (5.3)		
**Intentional beliefs to only use electronic PHRs when distrusting of partners (n=232)**					24.14	<.001
	Strongly agree/agree	93 (26.3)	62 (37.1)	31 (16.6)		
	Strongly disagree/disagree	139 (39.3)	47 (28.1)	92 (49.2)		
**Attitudinal belief of being suspicious of partners unwilling to share electronic PHR (n=270)^a^**					1.49	.22
	Strongly agree/agree	268 (75.7)	117 (68.9)	153 (81.8)		
	Strongly disagree/disagree	2 (0.6)	0 (0.0)	2 (1.1)		
**Preferred method for sharing a positive infection status (n=354)**					2.39	.49
	Using an electronic PHR	81 (22.9)	42 (25.2)	39 (20.9)		
	A conversation without electronic PHRs	158 (44.6)	68 (40.7)	90 (48.1)		
	Avoid sharing infection status	16 (4.5)	9 (5.4)	7 (3.7)		
	No preference	99 (28.0)	48 (28.7)	51 (27.3)		

^a^Scores between –1 and 1 for belief variables are not reported.

## Discussion

### Principal Results

Together, qualitative and quantitative findings offer several considerations for the potential role of electronic PHRs in facilitating STI health communication between partners. STI health communication is generally an important practice for our study population; however, whether and how discussions occur are functions of multiple individual and dyadic level factors. Inability to validate disclosed STI testing information, low personal agency for initiating discussions, nonawareness of STI testing talks as a health practice, and low discussion valuation related to partner-type or condom use may inhibit STI health communication from occurring. Electronic PHR access for STI screenings pose a viable solution to barriers preventing STI talks. Participants anticipate that access will be accompanied by testing discussions earlier in relationships and more frequently occurring prior to sexual encounters. Electronic PHRs are expected to add novel validation to screening information shared by partners and make it easier to initiate conversations. Male participants were more likely to believe electronic PHRs improve self-efficacy for discussions and in their ability to share positive results. Thus, electronic PHRs additionally offer new avenues for increasing male participation in STI prevention.

Self-reported behaviors potentiating STI transmission such as: sex without discussing STI testing, partner concurrency, sex while intoxicated, and condom-less sex practices is evidence of the need to continue targeting young black populations for STI interventions. Our study supports an increasing amount of burgeoning research on the feasibility and acceptability of delivering effective sexual health interventions through web- and mobile-based platforms [17,19,30-32]. Furthermore, it provides new insight into the role of patient electronic access in improving dyadic communication on STI risk. Participants almost ubiquitously anticipate that refusing to share STI screening results will warrant partner suspicion. These beliefs perhaps indicate that electronic PHR access will influence decisions related to sexual behavior.

### Limitations

This study has many strengths and limitations. Our findings offer rich data on electronic PHR access beliefs within our sample population in context of when, how, and why STI talks occur. The mixed-methods design allowed us to formatively identify important variables to study quantitatively for a novel practice. Similarly, the context of perceptions emerging from our study is largely in absence of prior participant exposure to electronic PHR access. Resources were not available for research assistants nor secondary coders; thus, qualitative findings lack inter-coder reliability and are therefore subject to researcher biases. Additionally, significant differences were observed in academic classification by gender in our non-random convenience sample of online survey participants.

Although we determined that many participants reported electronic PHR access would lead to earlier dyadic talks on STI prevention, future studies are needed to better understand whether electronic PHR access would truly extend proximity in time between STI talks and sexual encounters. Furthermore, to determine whether an increase in time between the two events minimizes the length of time to next STI screening between dyad members.

Low self-efficacy beliefs for sharing positive electronic results are likely an indicator of stigma associated with being diagnosed with an STI. Given the sample, our study does not provide substantial insight into perceptions about electronic PHR–facilitated STI talks among people with chronic infections like HIV and genital herpes. Nevertheless, reducing stigma and enabling individuals infected with STIs to safely and comfortably disclose infection status to partners remains an important challenge to prevention and care. Reducing stigma associated with discussing infection with partners may reduce behaviors that accompany non-disclosure of diagnosis, such as condom-less sex; in addition to stigma-related impacts on the HIV treatment cascade [33-36]. Future studies are needed to explore feasibility of addressing stigma-related outcomes with electronic PHRs.

### Conclusions

mHealth interventions incorporating electronic PHRs will offer new insight into strengthening infrastructure and the capacity to target disparities in STIs. Findings suggest that access to electronic PHRs for STI screening among subpopulations of black youth may improve both motivation and personal agency for initiating dyadic talks about testing. Results from this study will likely inform novel interventions that use access to electronic PHRs to stimulate important health-related discussions between sexual partners. The preventative capacity of electronic PHRs envisioned by our sample cannot be achieved without policies that support equipping them with patient portal access to STI screening records. Messages presented by healthcare providers on adopting electronic PHR–delivered STI results and electronic PHR–facilitated risk discussions will undoubtedly be key in adoption decisions. Moving forward requires studying strategies for implementing interventions that leverage electronic PHRs to create new sexual health communication channels with providers, peers, and family among black youth. With anticipated proliferation of electronic PHR adoption in generations to come, close attention is needed to ensure that black youth have equitable healthcare access to quality electronic PHR services [36].

## Abbreviations

CDC: Centers for Disease Control & Prevention
CMS: Centers for Medicare & Medicaid Services
DOI: Diffusion of Innovation Theory
eSHINE: Electronic Sexual Health Information Notification and Education
HITECH: Health Information Technology for Economic and Clinical Health Act
HSV-2: herpes simplex virus-2
IMBP: Integrative Model of Behavioral Prediction
MSM: men who have sex with men
PHR: personal health record
PI: principal investigator
STI: sexually transmitted infection
